# Identification of activation-tag *Arabidopsis* mutants with altered production of germination stimulants for *Phelipanche ramosa* (L.)

**DOI:** 10.1080/13102818.2014.911432

**Published:** 2014-07-31

**Authors:** Ina Kirilova, Iliya D. Denev, Rumyana Bineva, Maria Gevezova, Milena Alexandrova, Kaloyan Kostov, Rossitza Batchvarova

**Affiliations:** ^a^ Department of Plant Physiology and Molecular Biology, Plovdiv University, Plovdiv, Bulgaria; ^b^ Agricultural Academy, AgroBioInstitute, Sofia, Bulgaria

**Keywords:** activation-tag mutants, *Arabidopsis*, broomrapes, germination stimulants, *Phelipanche ramosa*

## Abstract

Germination of seeds of root parasites like broomrapes (*Orobanchaceae*) is tightly regulated by chemical products exuded from the roots of the host plant, known as germination stimulants (GSs). Changes in the levels of synthesis and emission of GS can allow the development of practical measures for control of the crops-harming parasitic species. However, the genes encoding enzymes responsible for GS biosynthesis are still unknown. We performed a large-scale screening of 62,000 *Arabidopsis* activation-tag mutants for alteration in susceptibility to *Phelipanche ramosa* and to identify lines with altered GS production among them. After five successive screenings we identified 36 lines with altered susceptibility to *P. ramosa*. Seven of them displayed altered levels of GS production. By using a combination of Southern blot and thermal asymmetric interlaced polymerase chain reaction (TAIL-PCR), we pinpointed the location of activation-tag constructs in these lines. A combination of differential display and quantitative real-time PCR (qRT-PCR) allowed us to identify several affected genes. Two of them are directly involved in isoprenoid biosynthetic pathway in chloroplasts, and we believe that their activation led to increased levels of GS production. We believe that these genes are responsible for increased GS production in five of the *Arabidopsis* lines resistant to *P. ramosa*.

## Introduction

Parasitism has evolved in at least 11 independent angiosperm lineages and now about 1% of all angiosperms (more than 4500 species) rely on other ‘host plants’ that provide them with the materials they cannot acquire from the abiotic environment.[1–3] Among them broomrapes demonstrate the highest level of adaptation: they are chlorophyll-lacking obligate root holoparasites that depend entirely on their hosts for nutrients, minerals and water.[4,5] To optimize their chances for survival, broomrapes have developed several mechanisms that ensure tighter coordination between the developmental stages of the parasite life cycle and that of the host plant.[3,6,7]. For instance, for germination to proceed, exposure of the ‘conditioned seed’ to exogenous xenognosins usually emitted in the host-root exudates is needed.[8–10] Thus, the germination of seeds of root parasites like broomrapes (*Orobanchaceae*) is tightly regulated by chemical recognition, i.e. by products exuded from the roots of the host plant, known as germination stimulants (GSs).[11] GSs originate from the chloroplast biosynthetic pathway and are mainly identified as strigolactones. Recently, Kohlen et al. [12] proposed that strigolactones may play the role of a new class of plant hormones that are involved not only in seed germination, but also in many other processes. For instance, the host–parasite exchange in chemical signals promotes the pre-symbiotic stage of the colonization of roots by arbuscular mycorrhizal fungi (AM fungi).[13,14] Parasitic weeds, like broomrapes, have probably evolved to take advantage of this signalling mechanism, and recognize the ‘chemical signature’ exuded by the prospective host plants.[12–15]

The genes encoding enzymes responsible for GS biosynthesis are still largely unknown, although the biogenetic origin of GS in tomato is thought to lie in the carotenoids pathway.[16–18] Deeper insight into the biochemical and genetic bases of production of these recognition molecules in host plants could throw more light on the mechanisms of co-evolution of parasites and their hosts and could aid the development of new practical measures for control of crop-harming parasitic species.[19]

*Arabidopsis* is an attractive model host plant for such studies, as it has been demonstrated to be a host for several *Orobanche* species.[20–22] Moreover, it has a relatively small and completely sequenced genome.[23]

One approach commonly used in the study of biochemical pathways on the genetic level is that based on gain-of-function mutations.[24] Such mutations can be induced through activation tagging,[24,25] i.e. with the aid of a T-DNA vector with four copies of the enhancer sequences from the constitutively active promoter of the cauliflower mosaic virus 35S (CaMV 35S) gene,[25] resulting in transcriptional activation of nearby genes.

Previously, we have demonstrated that activation tagging can produce *Arabidopsis* mutants with altered GS production.[26] The aim of this work was to perform large-scale screening of 62,000 *Arabidopsis* activation-tag mutants for alteration in GS production and to identify genes potentially affected by the activation-tag construct.

## Materials and methods

### Plant materials

Hemp broomrapes (*Phelipanche ramosa* L.) seeds were kindly provided by Dr. Tzveta Hristeva (Tobacco and Tobacco Products Institute, Bulgaria). Seeds were harvested from *P. ramosa-*infested tobacco fields in southern Bulgaria. The collection (N 31100) of 62,000 *Arabidopsis thaliana* T-DNA activation tag mutants, ecotype Col, was purchased from Nottingham Arabidopsis Stock Centre (NASC). Seeds arrived in pools of 100 to 300 lines per pool.

### Soil screening

Five successive screenings of *Arabidopsis* plants on soil infected with *P. ramosa* were used to select resistant genotypes. For this purpose *Arabidopsis* seeds were surface sterilized in an aqueous solution containing 2% active chlorine and 0.2% Tween 20. Seeds were carefully washed with sterile milli-Q water and placed on 1.5% Murashige and Skoog (MS) agar (2.2 g L^−1^ MS plant salt mixture, 15 g L^−1^ agar). The 19-cm Petri dishes with seeds were kept for four days in a refrigerator (4°C) to break dormancy and then were grown for two weeks in a growth cabinet (20°C, 14 h day/10 h night photoperiod) for two weeks. The seedlings were carefully transferred and planted in soil-containing multi-pot Araflats (51 positions, Arasystem 360 kit, # ASN 01). The soil was autoclaved and then mixed well with 50 mg L^−1^ *P. ramosa* seeds. The bottom holes of each pot were covered with synthetic fabric. Approximately 2 cm of the infested soil was poured in each pot and it was covered with about 3 cm of non-infested soil. The multi-pot Araflats were placed in Aratrays containing water to wet the soil and covered for a one-week adaptation period with humidity dome to keep the humidity high. Seedlings from each pool/line were planted as a control on non-infested soil in the same Arasystem kits. After adaptation, the immerging flowering stems of each *Arabidopsis* plant were placed in a conical holder (Aracon base) to collect seeds and a cylinder (Aracon tube) to prevent cross pollination. Plants were grown for 6–8 weeks in growth chambers (20°C, 14 h day/10 h night photoperiod, 80% relative humidity). The seeds of those plants that managed to flower approximately at the same time as the control plants were harvested for the next steps of the screening and then their roots were inspected under magnifying glass for attached parasitic plants. Only plants with roots free of any kind of *P. ramosa* attachments were considered potentially resistant and were subjected to subsequent successive screenings, using the same protocol.

### Collection of root exudates

Seeds of resistant *Arabidopsis* lines were surface sterilized and grown on ½ MS agar for 16–20 days as described above. Seedlings were carefully transferred in sterile multiwell plates (one plant per well) and roots were kept covered with 0.5 mL sterile milli-Q water for 24 h to collect root exudates. Before use, the collected exudates were diluted to equalize the root weight/volume ratio as described earlier.[26,27]

### Germination tests

Seeds of *P. ramosa* were surface sterilized as above. Seeds were carefully washed with sterile nano-pure water and placed between 1 cm glass fibre filter paper disks. These ‘sandwiches’ were moistened with sterile water and seeds were preconditioned at 26°C for 14 days, according to Mangnus et al. [28] with some modifications.[27] After preconditioning, the ‘sandwiches’ were removed from the conditioning Petri dishes and left in a sterile air steam until the surplus moisture was evaporated. Then they were placed in new Petri dishes and 0.1 mL of diluted root exudates were applied on double disks. The germination tests were carried out with root exudates collected from resistant lines and control wild-type *Arabidopsis* (Col) plants. Germination percentages were assessed after incubation for 8 d at 26°C. GR24 (0.4 mg L^−1^) was used as a positive control. Root exudates from non-host plants as well as sterile milli-Q water were used as negative controls. All experiments were conducted in 15 replicates.

### DNA isolation

Genomic DNA was extracted from 4 g fresh leaves collected from six-week-old plants of each resistant line and wild type Col –0. The cetyltrimethylammonium bromide (CTAB) protocol [29] was used.

### Thermal asymmetric interlaced polymerase chain reaction (TAIL-PCR)

TAIL-PCR was performed following the protocol adopted from Liu et al. [30,31] by using three specific nested primers (SP1 5′-TCCTGCTGAGCCTCGACATGTTGTC, SP2 5′-TCGACGTGTCTACATTCACGTCCA and SP3 5′-CCGTCGTATTTATAGGCGAAAGC) and three arbitrary highly degenerated primers (AD1: 5′-NTCGASTWTSGWGTT, AD2 5′-NGTCGASWGANAWGAA and AD3 5′-WGTGNAGWANCANAGA). A fourth nested primer (SP4: 5′-GGAGGAAAAGAAGAGTAATTA) was used for sequencing. The best results were achieved with the AD2 arbitrary primer. The TAIL-PCR reactions were carried out as previously described in details.[32] The PCR products were mixed with 4 μL of loading dye (Fermentas # R0611), loaded onto 1% agarose gel containing 0.5 mg L^−1^ ethidium bromide (final concentration) covered with 1X Tris-borate-EDTA (TBE) buffer and separated by applying 7 V cm^−1^ electrical current. The size of the products was determined by comparison with a DNA ladder (Fermentas GeneRuler # SM0311). The PCR products were visualized by ultraviolet (UV) light and were isolated from the agarose by cutting out with a clean surgical blade. Then they were extracted with a QIAquick Gel extraction kit (Qiagen, # 28704) following the original protocol. The purified products were sequenced by GATC Biotech AG (Cologne, Germany), using the SP4 primer.

### Southern blot

For restriction digestion 2.8 μg of gDNA were taken from each sample and incubated with restriction enzyme EcoRI (Amersham Pharmacia Biotech Inc.) overnight at 37°C (according to the manufacturer's instructions). DNA fragments were separated by electrophoresis in 1% ethidium-bromide-free (EtBr-free) agarose gel in 1x Tris-acetate-EDTA (TAE) buffer (3.75 mmol L^−1^ Tris, 1 mol L^−1^ ethylenediaminetetraacetic acid (EDTA), 2 mmol L^−1^ sodium acetate) overnight at 20–25 V in a cold room. After standard treatment steps for Southern blot with a digoxigenin (DIG)-labelled probe,[33] the samples were transferred on BrightStar®-Plus Positively Charged Nylon Membrane (Invitrogen) and fixed on it by UV-crosslinking (100 mJ cm^−2^). The phosphinothricin (*BAR*) resistance gene, which is part of the pSKI15 vector, was used to design the hybridization probe. A combination of two gene-specific PCR primers (BAR Fw: 5′-ATATTCATTAGAATGAACCGAAACC-3′ and BAR Rev: 5′-GACTCTAGCGAATTCCTCGAGTAT-3′) was used to isolate a 810 bp fragment of the *BAR* gene. It was used as a template in combination with PCR DIG Probe Synthesis Kit (Roche) to generate the hybridization probe labelled with DIG-dUTP (Dig-11-dUTP-labelled *BAR* probe) in PCR. A 1:4 ratio of DIG-dUTP to dTTP was used. The following PCR programme was used for synthesis of the DIG-labelled probe: initial denaturation 94°С/3 min; followed by 30 cycles of 94°С/45 s, 58°С/45 s and 72°С/90 s. After a final extension of 5 min at 72°C, the PCR reactions were immediately cooled to 4°С. The probe was purified from the reaction mix by a Qiagen PCR purification kit and stored shortly at 4°C. All steps of prehybridization of the membrane and hybridization were performed according to the protocol of the manufacturer, using DIG Easy Hyb (Roche). The hybridization was carried out overnight at 42°C using 15 mL of hybridization buffer (DIG Easy Hyb, Roche) containing 20 ng mL^−1^ of DIG-labelled probe. The membrane was washed twice from the hybridization solution with 2х saline-sodium citrate (SSC)/0.1% sodium dodecyl sulphate (SDS) at room temperature and twice in 0.2х SSC/0.1% SDS. CSPD ready-to-use (Roche) luminescent detection kit was used to manifest numbers of inserts in each line. The manufacturer protocol was followed. Luminescence was detected using Amersham film plates (Amersham Pharmacia Biotech Inc.) and exposition time between 1:30 and 2:15 h.

### RNA isolation

RNA was isolated from root tissue collected both from resistant and wild-type plants. The samples were frozen and ground in liquid nitrogen. Total RNA was isolated from 50 mg of frozen tissue by RNeasy plant mini kit (Qiagen). The protocol provided by the supplier was followed, including the DNase treatment.

### Differential display

Differential display procedures were carried out essentially as described by Liang and Pardee.[34] Total RNA (3 μg) was reverse transcribed in a final volume of 25 μL using the Superscript II reverse transcriptase (Life Technologies) and four different anchored primers oligo(dT11)MN (MN = AG, AC, CC or GC), following the protocol of the enzyme supplier. Reactions were diluted (1:60) and 1 μL aliquots were used as template for PCR. Differential display PCR was carried out in 25 μL volume containing the same oligo(dT11)MN anchored primer and one of the arbitrary primers (AP1: 5′-CAGGCCCTTC; AP2: 5′- TGCCGAGCTG; AP3: 5′-AGTCAGCCAC; AP4: 5′-AATCGGGCTG; AP5: 5′-AGGGGTCTTG; AP6: 5′-GGTCCCTGAC; AP7: 5′-GAAACGGGTG; or AP8: 5′-GTGACGTAGG). After initial denaturation for 4 min at 94°C, 30 cycles of amplification were conducted for 30 s at 94°C; 2 min at 40°C and 45 s at 72°C followed by an additional extension period for 5 min at 72°C. The PCR products were separated by electrophoresis in a 7% denaturing polyacrylamide gel. After electrophoresis the gel was washed with ddH_2_O and the products were visualized using silver staining.[35] The differential display procedures were repeated five times independently as suggested by Stein and Liang [36] to reduce the number of false positives due to reverse transcription and PCR artefacts. Bands demonstrating consistent increasing or decreasing of expression patterns were considered as differentially expressed genes. Bands of interest were cut out from the gel and products recovered.[35] The products were re-amplified with the same primer sets, purified and A/U cloned in pDrive vector for sequencing.

### Quantitative real-time PCR (qRT-PCR)

An applied Biosystems model 7500 qRT-PCT apparatus was used. Primers for the qRT-PCR analysis were designed to accommodate exon-intron junction at 3′ side in order to minimize effect of any genomic DNA contaminations. All reactions contained 10 μL of SYBR Green Master Mix (Applied Biosystems), 25 ng of cDNA, and 200 nmol L^−1^ of each gene-specific primer in a final volume of 20 μL. The qRT-PCR programme included: 50°C for 2 min, 95°C for 10 min, followed by 40 cycles of 95°C for 15 s and 60°C for 1 min.

### Bioinformatics

The positions of T-DNA activation constructs were determined by using online NBLAST [37] of the sequences rescued by TAIL-PCR against the TAIR Arabidopsis database. The same approach was used in the identification of differentially expressed genes.

## Results and discussion

### Screening of T-DNA activation-tag *Arabidopsis* mutants

The first screening of *Arabidopsis* activation-tag mutant lines for selection of individual genotypes resistant to the broomrape *P. ramosa* included all 62,000 mutant lines. The development synchronization was achieved by germinating *Arabidopsis* seeds on ½ MS agar (see Figure S1 in the Online Supplemental Appendix). This step was included because different *Arabidopsis* mutants need various times to germinate and form seedlings that are strong enough to be transplanted on soil. Many potentially promising genotypes were lost during preliminary experiments when we used direct sowing on soil.[26] Another important feature of this screening was that each individual plant was initially planted in a separate pot of 150-position multi-pot flats. In total 742,658 seedlings were planted and grown on infected soils. The guiding idea behind this was the expected effect of the root parasite on the growth and development of the host. As the parasite withdraws water and photosynthetic products from the host, it substantially retards the development of such tiny plants like *Arabidopsis*. Therefore, only tolerant and resistant genotypes can grow approximately as fast as control plants. This concept was proved in our preliminary experiments.[26,38,39] These differences became apparent after a few weeks of growth, and the promising plants were transplanted to wider 45-position Araflats and immediately isolated from other plants by Aracon-base and Aracon-tube (see Figure S2 in the Online Supplemental Appendix). This not only prevented crosspollination, but also limited spreading of diseases by insects. The developmental rates of experimental plants from each pool were compared with those of control plants unchallenged with *P. ramosa* from the same batch. As a result of the first screening, 3435 individual plants managed to form flowers and seeds within 8–10 weeks just as control plants. Their seeds were collected and roots inspected for attached parasitic plants (see Figure S3 in the Online Supplemental Appendix). After root inspections, the number of potentially resistant plants was further limited to 1218. Their seeds were further challenged by increasing concentrations of *P. ramosa* seeds in the soil during the next four successive cycles of screening. As a final result of the screening we succeeded to identify 36 individual lines originating from four initial pools of lines, which demonstrated consistent resistance against *P. ramosa* parasitation.

Several mechanisms and processes could be involved in resistance against root parasite ranging from alteration of GS production to hypersensitive response to parasitization resulting in programmed cell death, leading to excision of affected root branches. In our investigation we were particularly interested in mutant lines with altered GS production. The existing instruments are still not sensitive enough to detect and determine directly the presence of GS emitted by a few tiny plants. Therefore, we compared the GS production in different resistant lines by a germination test originally designed by Magnus et al. [28] and modified for the needs of the *Arabidopsis*–*Orobanche* system by Denev et al.[26]

### Testing resistant lines for alteration in GS production

**Figure 1.**
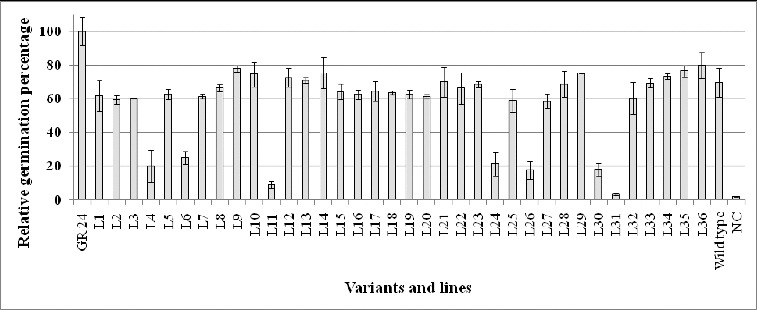
Relative germination of *P. ramosa* seeds incubated with root exudates from *Arabidopsis thaliana* activation-tag mutants potentially resistant to *P. ramosa*.

The experiments were performed as described by Denev et al. [26] with a few differences: from our previous expertise we know that the ability of broomrape seeds to germinate experience seasonal variations. That is why, all experiments were performed during the spring/early summer of 2012. The results initially were obtained as percentage of germinated vs. total number of *P. ramosa* seeds treated with root exudates. Because germination depends not only on GS production but also on various conditions, the results were compared with the percentage of germinated sees after treatment with GR24 (0.4 mg L^−1^ – positive control). The percentage of germination after treatment with GR 24 ranged between 65% and 83%. In order to achieve comparability between different replicas, we accepted provisionally that germination in the positive control is 100% and all results were recalculated accordingly. The mean values of all fifteen replicas are presented in Figure 1.

The results revealed that seven of the lines (L4, L6, L11, L24, L26, L30 and L31) provoked much lower germination in comparison with other lines (Figure 1). It is known that GSs have an optimal effect on the germination of broomrape seeds in concentrations ranging between 10^−7^ and 10^−9^ mol L^−1^. Concentrations of GS below or above these values suppress seed germination.[26] Therefore, to test whether the observed differences are due to either overproduction or much lower GS production, we performed germination tests with much greater dilutions of root exudates. The obtained results showed that except L11 and L31, the other five lines most probably overproduce GS. One alternative explanation could be the overproduction of a hypothetical inhibitor of broomrape seed germination. Since such compound(s) have not been found yet, this explanation is less probable.

The resistant mutant lines have levels of GS production similar to the wild type plants. Therefore, it could be assumed that their resistance is based on mechanisms other than changes in GS production.

### Determination of position and number of T-DNA inserts

Total DNA was isolated from the 36 mutant lines. The quantity of the isolated DNA was identified spectrophotometrically and ranged from 180 to 310 ng μL^−1^. The quality of the isolated DNAs was determined by gel electrophoresis which proved that all DNA samples contain highly polymeric DNA without RNA contaminations (result not shown).

**Figure 2.**
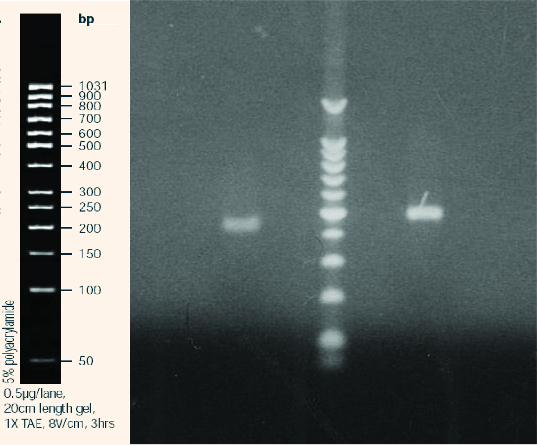
Electrophoretic separation (1% agarose gel) of the hybridization probe labelled with Dig-11-dUTP (right) as compared to the control (left).

**Figure 3.**
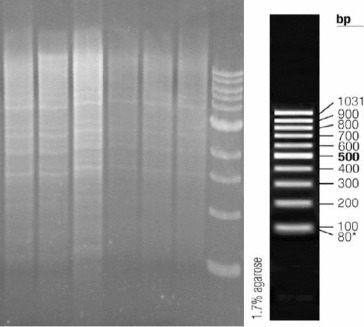
Electrophoretic separation (1% agarose gel) of genomic DNA digested with EcoRI. From left to right: L4, L12, L18, Wt, L21 and L36.

Initially, the number of T-DNA insertions in the genomes of each line was determined. For this purpose, we used Southern blot hybridization with a Dig-11-dUTP–labelled *BAR* probe (Figure 2). The isolated genomic DNA was digested with EcoRI and separated by electrophoresis in 1% EtBr-free agarose gel (Figure 3).

**Figure 4.**
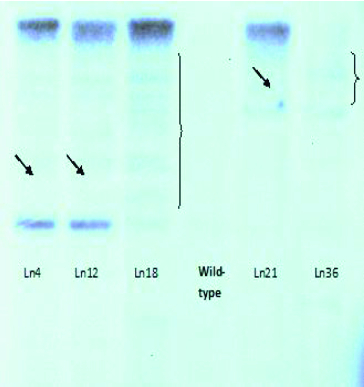
Southern blot hybridization with genomic DNA isolated from activation-tag mutants of *Arabidopsis* lines resistant to *P. ramosa*.

The Southern blot hybridization analysis was repeated four independent times. The results indicated that the resistant plants of 32 lines have a single copy of the *BAR* gene in their genome and, respectively, of the T-DNA activation insert (Figure 4). The analyses of plants from the other four lines did not give unambiguous results. We assume that in two of these lines the insert was presented in more than one copy (Figure 4), while the other two lines did not display a hybridization signal. These four genotypes were not subjected to TAIL-PCR because we did not expect reliable results. The seven genotypes with altered GS production were among the 32 genotypes with a single activation T-DNA construct.

The positions of the activation T-DNA constructs into the genome of these genotypes were determined by TAIL-PCR. The three successive PCR reactions with nested and arbitrary primers resulted in amplification of a single TAIL-PCR product per line. These products were isolated and sequenced and the obtained sequences were simultaneously analysed using the NBLAST algorithm versus the NCBI and TAIR genetic databases.

The sequence data allowed us to unambiguously identify the positions of T-DNA activation tag insertions in *Arabidopsis* lines genomes in all seven genotypes with altered GS production. The results are presented in Table 1.

**Table 1.** List of identified genes affected by T-DNA activation-tags in mutant *Arabidopsis* lines with altered GS production.

Line	Gene	Expected effect
L4	At3g22180	Knock-out
L6	At3g47090	Knock-out
L11	At3g29110	Activation
L24	At5g23960	Activation
L26	At5g23960	Activation
L30	At5g23960	Activation
L31	At5g23960	Activation

In lines 24, 26, 30 and 31 the T-DNA inserts are at approximately the same location. This is not surprising, since GS production is not likely to be influenced by a huge amount of genes. Repetition of certain locations actually increases the likelihood that the affected genes actually contribute to increased GS production. In two of the lines (L4 and L6) the activation T-DNA insertions are in the coding sequence of genes At3g22180 and At3g47090. This potentially means that these genes are knocked-out because the insert has a length of approximately 10 kB and several terminator sequences which block the expression. However, the neighbouring genes can still be activated. It has been reported many times that not always the genes in close proximity to the T-DNA activation insert are those which relate to the observed phenotype. The insert can, for instance, activate distant genes localized even in other chromosomes through stimulation of regulatory factors.[24,40] Therefore, we attempted to identify all affected genes by means of transcriptomics.

### Transcriptomics analyses: differential display and qRT-PCR

**Figure 5.**
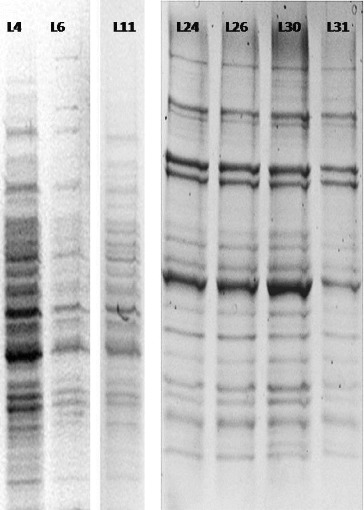
Separation of differential display products (7% polyacrylamide gel).

Plants for transcriptomics analyses were grown under sterile conditions (see Figure S4 in the Online Supplemental Appendix) to prevent any gene activation due to bacterial or fungal infections. The obtained differential display products (Figure 5) were isolated and sequenced. In order to make a more precise determination of the expression levels of affected genes, qRT-PCR was used, as recommended by a number of authors. Combined results are shown in Table 2.

**Table 2.** List of identified genes with altered expression levels.

Line	Affected gene*	Putative function
4	At3g22180 (down)	The gene encodes DHHC-type zinc finger family protein; FUNCTIONS IN: zinc ion binding; INVOLVED IN: biological_process unknown; LOCATED IN: plasma membrane; EXPRESSED IN: 9 plant structures; EXPRESSED DURING: 6 growth stages; CONTAINS InterPro DOMAIN/s: Zinc finger, DHHC-type (InterPro:IPR001594)
	At3g22183 (up)	The gene encodes unknown protein; LOCATED IN: endomembrane system
	At3g22190 (up)	The gene encodes IQ-domain 5 protein (IQD5); FUNCTIONS IN: calmodulin binding; INVOLVED IN: biological_process unknown; LOCATED IN: nucleus and plasma membrane; EXPRESSED IN: 24 plant structures; EXPRESSED DURING: 13 growth stages; CONTAINS InterPro DOMAIN/s: IQ calmodulin-binding region (InterPro:IPR000048)
6	At5g47090 (down)	Gene encodes unknown protein; CONTAINS InterPro DOMAIN/s: Protein of unknown function DUF2052, coiled-coil (InterPro:IPR018613)
	At5g47100 (up)	The gene encodes a member of AtCBLs (Calcineurin B-like Calcium Sensor Proteins. CBL9 interacts with and targets CIPK23 to the plasma membrane in vivo in response to water deprivation
11	At3g29110 (up)	Nuclear encoded terpenoid synthase/cyclase, localizes in plastids in roots and plant sperm cell
24	At5g23960 (up)	The gene encodes a sesquiterpene synthase involved in generating all of the group A sesquiterpenes. Localized in chloroplasts. Response to herbivores and pathogens
26	At5g23960 (up)	The gene encodes a sesquiterpene synthase involved in generating all of the group A sesquiterpenes. Localized in chloroplasts. Response to herbivores and pathogens
30	At5g23960 (up)	The gene encodes a sesquiterpene synthase involved in generating all of the group A sesquiterpenes. Localized in chloroplasts. Response to herbivores and pathogens
31	At5g26749 (up)	C2H2 and C2HC zinc fingers superfamily protein; FUNCTIONS IN: zinc ion binding, nucleic acid binding; INVOLVED IN: biological_process unknown; LOCATED IN: cellular_component unknown
	At5g23960 (up)	The gene encodes a sesquiterpene synthase involved in generating all of the group A sesquiterpenes. Localized in chloroplasts. Response to herbivores and pathogens

*(up) indicates expression levels elevated more than fivefold; (down) indicates little or no expression detected.

The results for the expression of the affected genes highlighted several interesting groups of genes. In three of the lines (L24, L26 and L30), the elevated production of GSs is associated with activation of nuclear-encoded, chloroplast localized E-beta-caryophyllene synthase (At5g23960). It is known that the chloroplast terpenoid pathway is the source of GSs which have a chemical structure of sesquiterpene lactones.[16,38] The activated gene is involved in the biosynthetic pathway of lactones. Previous investigations have shown that the gene is activated in response to attack by phytophagous insects and phytopathogens.[41] All this gives us reason to believe that we have discovered a gene directly involved in the biosynthesis of GS. Surprisingly, line 31 displayed a significant increase in the expression of gene At5g26749. It encoded a product that belongs to the C2HC zinc-finger protein superfamily binding to nucleic acids. It could be a regulatory factor whose specific involvement in biological processes is still unknown.

The second equally promising gene is identified by line 11, in which upregulation of a nuclear encoded, chloroplast-localized terpenoid cyclase was observed. This is another protein involved in the chloroplast terpenoid pathway, suggesting that most probably At3g29110 is a second gene involved in GS production.

The knocking out of genes At3g22180 (L4) and At3g47090 (L6) was confirmed. However, a few other genes are activated. The relation between GS production and affected genes in these two lines is under elucidation.

## Conclusions

After screening of 62,000 activation tag lines, seven lines with altered production of germination signals were identified. The data from TAIL-PCR and qRT-PCR pointed to two very promising genes involved in isoprenoid biosynthesis in the chloroplast. Because GSs originate from chloroplasts we believe these genes are responsible for increased GS production. We will focus our future work, particularly searching for similar genes in crops parasitized by broomrapes, which in term can lead to creation of crop varieties resistant to infestation of broomrapes. 

## Supplemental data and research materials

Supplemental data for this article can be accessed at here.
